# Associations of Serum 25-Hydroxyvitamin D, Parathyroid Hormone and Calcium with Cardiovascular Risk Factors: Analysis of 3 NHANES Cycles (2001–2006)

**DOI:** 10.1371/journal.pone.0013882

**Published:** 2010-11-09

**Authors:** Abigail Fraser, Dylan Williams, Debbie A. Lawlor

**Affiliations:** Department of Social Medicine, Medical Research Council Centre for Causal Analyses in Translational Epidemiology, University of Bristol, Bristol, United Kingdom; Maastricht University, Netherlands

## Abstract

**Background:**

Increasing evidence suggests a role for mineral metabolism in cardiovascular disease risk. 25-hydroxyvitamin D (25(OH)D), parathyroid hormone (PTH), and calcium may be directly associated with cardiovascular risk factors or mediated by each other.

**Methodology/Principal Findings:**

We combined data for adult participants in three cycles of the National Health and Nutrition Examination Survey (2001–2, 2003–4, 2005–6), a representative sample of the civilian, non-institutionalized US population (N = 3,958). Using this data we examined joint associations of 25(OH)D, PTH and calcium with a range of cardiovascular risk factors. 25(OH)D was inversely associated with fasting insulin (mean difference in insulin per 1 standard deviation 25(OH)D: −0.053 (95%CI: −0.091, −0.015)), glucose (−0.046 95%CI: −0.081, −0.012) and systolic blood pressure (SBP) (−0.032 95%CI: −0.062, −0.001), and positively associated with high density lipoprotein cholesterol HDL-c (0.088 95%CI: 0.044, 0.148), after adjustment for ethnicity, smoking, socio-economic status and waist circumference. PTH was positively associated with diastolic blood pressure (0.110, 95%CI: 0.055, 0.164) in confounder adjusted models, but was not associated with other cardiovascular risk factors. Albumin adjusted calcium was associated with triglycerides (0.102 95%CI: 0.063, 0.141), postload glucose (0.078, 95%CI: 0.025, 0.130), fasting insulin (0.074, 95%CI: 0.044, 0.104), HbA1c (0.070, 95%CI: 0.036, 0.105), SBP (0.064, 95%CI: 0.028, 0.100), fasting glucose (0.055, 95%CI: 0.018, 0.092) and low density lipoprotein cholesterol (0.052, 95%CI: 0.014, 0.091). With mutual adjustment for each other, these associations remained essentially unchanged.

**Conclusions/Significance:**

Lower levels of 25(OH)D and higher levels of calcium and PTH appear to be associated with different cardiovascular risk factors and may therefore affect cardiovascular disease risk through different mechanisms.

## Introduction

Increasing evidence suggests a role for mineral metabolism in cardiovascular disease risk. Lower levels of 25-hydroxyvitamin D (25(OH)D,[1]–[3] and higher levels of parathyroid hormone (PTH)[4] and calcium[5] have been associated with increased risk of cardiovascular disease events in prospective cohort studies, but the mechanisms underlying these associations are unclear. There is evidence that lower levels of 25(OH)D and higher levels of PTH are associated with cardiovascular risk factors, including hyperglycaemia, insulin resistance, type 2 diabetes, dyslipidaemia and high blood pressure,[5]–[12] but to our knowledge no previous study has examined associations of all three of 25(OH)D, PTH and calcium with cardiovascular risk factors to.

Levels of 25(OH)D, PTH and calcium are interdependent. 25(OH)D is a key regulator of circulating calcium levels and of bone absorption of calcium,[13] and via its action on circulating calcium levels, indirectly affects PTH levels. PTH is produced by the parathyroid gland, primarily in response to low calcium levels, and acts via several mechanisms to increase circulating blood calcium levels.[14] In addition, the hydroxylation of 25(OH)D in the kidneys to 1,25-dihydroxycholecalciferol (the active form of vitamin D) is tightly regulated by plasma PTH and serum calcium levels.[13] Thus, levels of 25(OH)D, PTH, and calcium may be directly associated with cardiovascular risk factors or mediated by each other.

The objectives of the current study were (i) to examine the association of 25(OH)D, PTH and calcium with a range of cardiovascular risk factors; (ii) to examine whether associations of 25(OH)D, PTH and calcium with risk factors are independent of each other or not.

## Methods

NHANES is a complex, multistage probability sample of the healthy, ambulatory population of the U.S. Full details on survey methods can be found at http://www.cdc.gov/nchs/nhanes.htm. Data on adults (≥20 years old) from three surveys: 2001–2, 2003–4 and 2005–6 were combined and are included here.

### Ethics Statement

Ethical approval for the study was obtained from the NCHS Research Ethics Review Board (ERB) and further ethical approval for use of NHANES data that is freely available on the web is not required as it is anonymized. Written consent was obtained from participants.

Information on age, ethnicity, physical activity, smoking, alcohol intake in the past month, family income, diabetes and CHD status (using the Rose angina questionnaire[15]) were self-reported. A poverty-income ratio (PIR) was calculated for each family based on self-reported family income in relation to poverty threshold, family size and calendar year. Values below 1 are below the official poverty threshold while PIR values of 1 or greater indicate income above the poverty level (range 0–5). Smoking was coded as never, ex, currently on some days and currently every day. Detailed information on leisure time physical activities in the past month was recorded and assigned a metabolic equivalent (MET) score. Information on supplementation during the past 30 days was collected and participants were asked to present the container. Vitamin D intake from supplementation was calculated per participant according to the brand of supplementation taken.

Participants underwent a medical examination in which weight, standing height and waist circumference were measured in a standardized fashion. Seated resting blood pressure was measured by a physician and calculated as the average of available measurements (min 1 max 4), excluding the first measurement if more than one was available.

Blood samples were immediately centrifuged, aliquoted and frozen at −70°C. Samples were shipped on dry ice to central laboratories and stored at −70°C. In all three cycles, 25(OH)D was measured using a Diasorin RIA method. PTH was measured with an automated analyzer using a sandwich principle in both 2003–2004 and 2005–2006. Calcium was measured using the LX20 system that uses an indirect (or diluted) ISE methodology. The method used to measure the albumin concentration on the LX20 is a bichromatic digital endpoint method. We calculated albumin adjusted calcium using the formula: adjusted calcium (mmol/l)  =  measured total Ca (mmol/l) +0.02 (40− serum albumin [g/l]).

High density lipoprotein cholesterol (HDL-c) was measured by direct immunoassay. As the assay method was changed in 2003, we used HDL-c data from the 2003–4 and 2005–6 surveys only. Triglycerides were measured using a timed-endpoint method. Low density lipoprotein cholesterol (LDL-c) levels were derived on examinees that were examined in the morning session only, according to the Friedewald calculation, which uses information on total-, HDL-c and triglycerides.

Fasting insulin, glucose and triglycerides were available for a random subgroup of participants who attended the morning clinics. Fasting insulin was measured in 2000–2001 using a radioimmunoassay, a two-site immunoenzymometric assay in 2003–2004 and ELISA in 2005–2006. Linear regression models were used to adjust 2001–2002 and 2005–2006 values to 2003–4 values, as recommended by the National Center for Health Statistics[16]. HOMA-R was calculated as the product of fasting glucose (mmol/l) and insulin (µU/ml) divided by the constant 22.5.[17] Plasma glucose was measured using an enzyme hexokinase method in all included surveys and the 2005–2006 values (Hitachi 911) were corrected to the (Roche Cobas Mira method) 2003–2004 values. Serum creatinine was measured with the Jaffe rate method and measures from the 2005–2006 survey were corrected according as recommended. Glomerular filtration rate (GFR), a measure of kidney filtration function was estimated with the abbreviated Modification of Diet in Renal Disease study equation.[18] An oral glucose test was added to the survey protocol in 2005–2006 and the fasting and 2 hour postload values from this glucose tolerance test are used in analyses presented here.

### Statistical Analysis

All analyses were adjusted for sampling probability (via weights) and cluster effects using the **svy** procedures in Stata version 10. Measurements for fasting insulin, HOMA-R, triglycerides and PTH were naturally log transformed to normalize distributions. Fasting insulin and HOMA-R are nearly perfectly correlated (Pearson's r = 0.97). We report result for fasting insulin, however all results are the same if fasting insulin is replaced with HOMA-R. Linear regression was used to describe the distribution of participant characteristics across fifths of the 25(OH)D distribution, the adjusted calcium and the (logged) PTH distribution. In multivariable analyses, 25(OH)D, logged PTH, calcium and risk factors were all z-scored, so that coefficients between each are directly comparable. In these analyses we adjusted for the following potential confounders: age, sex, ethnicity, PIR, waist circumference (or BMI), smoking and physical activity. In the final model the associations of each exposure with risk factors were mutually adjusted for the remaining two. Since 25(OHD, PTH and calcium may act via mediating effects and/or be part of the same causal mechanism we considered the confounder adjusted models (before mutual adjustment for each other) to be our best estimate of the unconfounded association of each with cardiovascular risk factors.

All analyses presented here include data for a random subgroup of participants who attended the morning clinic and provided fasting blood samples (N = 6,695). Plasma 25(OH)D was available for 6,616 and calcium (and albumin) for 6,614 (99%). We excluded pregnant women (N = 402), participants with a history of diabetes (N = 758) and angina (N = 155), leaving 5,343 eligible participants. Of these, complete data on sex, age, ethnicity, PIR, smoking, BMI, waist circumference, blood pressure, LDL-c, HbA1c, fasting glucose, insulin and triglycerides was available for 3,958 participants (74% of eligible). These participants formed our main analysis sample for analyses with 25(OH)D. PTH was only measured in two of the three NHANES cycles used in these analyses (2003–2004 and in 2005–2006) and therefore the analysis sample available for PTH is 2,554 (75% of those eligible in these two surveys). Physical activity and alcohol data was additionally available for 66–67% of the analysis samples for 25(OH)D and PTH. We did not limit analyses to participants with physical activity and alcohol data, but compared results for the subgroups with and without these data.

HDL-c was available for 2,564 participants in the 2003–4 and 2005–6 surveys only and 2-hour postload glucose was available for 1,193 participants in the 2005–6 survey only. For these risk factors analyses are limited to these numbers. We also performed sensitivity analyses excluding participants with osteoporosis (N = 202) and those reporting taking vitamin D supplementation (N = 1,457), excluding participants with fasting glucose values greater than 7 mmol/l (N = 121) and excluding participants with an estimated GFR <60 mL/min/1.73 m^2^ (N = 239).

## Results

Amongst the 3, 958 participants who formed our main analysis sample weighted mean 25(OH)D was 59.6 nmol/l (SE = 1.01) and for adjusted calcium it was 2.31 mmol/l (SE = 0.002). Weighted geometric mean PTH among the 2,554 from the two surveys where this was assayed was 38.0 ng/l (SE logged PTH = 0.01). 25(OH)D was modestly inversely associated with log PTH (Pearson's r = −0.28, p-value <0.001) but was not associated with adjusted calcium (Pearson's r = −0.01, p-value = 0.49). Log PTH and adjusted calcium were weakly inversely associated (Pearson's r = −0.08 p-value = 0.001).

Participant characteristics across fifths of the 25(OH)D distribution, the PTH distribution and the adjusted calcium distribution are given in Table S1.

Table 1 presents the multivariable analysis of associations of 25(OH)D with cardiovascular risk factors. In age and sex adjusted models (model 1) there was strong statistical evidence of association of 25(OH)D with all risk factors except for diastolic blood pressure (DBP) and LDL-c. For all associations lower 25(OH)D levels were associated with more adverse cardiovascular risk factor levels. All associations persisted after controlling for ethnicity, PIR and smoking (model 2). Inverse associations with systolic blood pressure (SBP), HbA1c, fasting glucose and fasting insulin, and the positive association with HDL-c also remained when adding waist circumference to the models (model 3), but inverse associations with triglycerides and 2-h postload glucose were attenuated to the null.

**Table 1:** Multivariable associations of 25(OH)D with cardiovascular risk factors.

	SD	Model 1 (N = 3,958)		Model 2 (N = 3,958)		Model 3 (N = 3,958)		Model 4 (N = 2,554)	
		Mean change z-score per 1 z-score difference in 25(OH)D (95%CI), p-value							
SBP (mm/Hg)	19.20	−0.077 (−0.107, −0.046)	<0.001	−0.058 (−0.091, −0.025)	0.001	−0.032 (−0.062, −0.001)	0.04	−0.029 (−0.065, 0.007)	0.11
DBP (mm/Hg)	11.52	−0.028 (−0.071, 0.014)	0.19	−0.035 (−0.083, 0.013)	0.18	−0.002 (−0.049, 0.045)	0.97	0.028;(−0.034,0.090)	0.36
Fasting glucose (mmol/l)	0.80	−0.082 (−0.118, −0.047)	<0.001	−0.089 (−0.128, −0.050)	<0.001	−0.046 (−0.081, −0.012)	0.008	−0.050;(−0.093,−0.007)	0.03
2-h glucose† (mmol/l)	0.37	−0.005 (−0.007, −0.002)	<0.01	−0.004 (−0.007, −0.001)	0.02	−0.001 (−0.004, 0.002)	0.64	−0.000 (−0.003, 0.003)	0.89
HbA1c (%)	0.49	−0.112 (−0.136, −0.087)	<0.001	−0.063 (−0.089, −0.036)	<0.001	−0.022 (−0.046, 0.001)	0.06	−0.021 (−0.049, 0.008)	0.16
Fasting insulin‡ (pmol/l)	0.80	−0.193 (−0.234, −0.151)	<0.001	−0.182 (−0.224, −0.140)	<0.001	−0.053 (−0.091, −0.015)	0.007	−0.061 (−0.105, −0.017)	0.009
Fasting triglycerides‡ (mmol/l)	0.51	−0.039 (−0.077, 0.000)	0.05	−0.081 (−0.121, −0.040)	<0.001	−0.014 (−0.056, 0.028)	0.49	−0.043 (−0.095, 0.008)	0.10
HDL-c* (mmol/l)	0.40	0.136 (0.076, 0.195)	<0.001	0.155 (0.099, 0.212)	<0.001	0.088 (0.034, 0.141)	0.002	0.102;(0.048,0.155)	0.001
LDL-c (mmol/l)	0.93	0.008 (−0.030, 0.045)	0.67	0.007 (−0.033, 0.048)	0.71	0.032 (−0.010, 0.074)	0.13	0.005;(−0.048,0.058)	0.85

SBP – systolic blood pressure; DBP – diastolic blood pressure.

Model 1 – age and sex adjusted.

Model 2 – as model 1 plus ethnicity, PIR, smoking.

Model 3 – as model 2 plus waist circumference.

Model 4 – as model 3 plus PTH and adjusted calcium.

*N = 2,557 in models 1 to 3, and N = 2,554 in model 4.

† N = 1,193 in all models.

‡ z-score of log transformed values.

Associations of PTH with cardiovascular risk factors are presented in Table 2. In age and sex adjusted models PTH was positively associated with DBP and SBP, HbA1c, fasting and postload glucose, and fasting insulin. Age and sex adjusted associations with HbA1c and postload glucose were attenuated to the null after adjustment for ethnicity, PIR and smoking (model 2). Further adjustment for waist circumference attenuated associations with SBP, HDL-c and fasting glucose to the null, so that the only association remaining was that of the positive association of PTH with DBP (model 3).

**Table 2 pone-0013882-t002:** Multivariable associations of PTH with cardiovascular risk factors.

	Model 1 (N = 2,554)		Model 2 (N = 2,554)		Model 3 (N = 2,554)		Model 4 (N = 2,554)	
	Mean change z-score per 1 z-score difference in 25(OH)D (95%CI), p-value							
SBP (mm/Hg)	0.089 (0.026, 0.153)	0.007	0.077 (0.011, 0.143)	0.02	0.049 (−0.016, 0.113)	0.13	0.055 (−0.004, 0.115)	0.07
DBP (mm/Hg)	0.154 (0.010, 0.211)	<0.001	0.150 (0.089, 0.211)	<0.001	0.110 (0.055, 0.164)	<0.001	0.128 (0.069, 0.188)	<0.001
Fasting glucose (mmol/l)	0.051 (0.002, 0.100)	0.04	0.048 (−0.007, 0.103)	0.08	−0.004 (−0.055, 0.047)	0.88	−0.007 (−0.055, 0.041)	0.76
2-h glucose* (mmol/l)	0.155 (0.077, 0.234)	0.01	0.128 (0.038, 0.218)	0.17	0.042 (−0.044, 0.128)	0.47	−0.000 (−0.003, 0.003)	0.89
HbA1c (%)	0.058 (0.015, 0.101)	0.01	0.032 (−0.015, 0.079)	0.17	−0.016 (−0.058, 0.026)	0.45	−0.008 (−0.049, 0.033)	0.70
Fasting insulin† (pmol/l)	0.197 (0.134, 0.259)	<0.001	0.173 (0.106, 0.241)	<0.001	0.022 (−0.276, 0.071)	0.38	0.023 (−0.017, 0.063)	0.25
Fasting triglycerides† (mmol/l)	0.021 (−0.040, 0.083)	0.48	0.044 (−0.021, 0.109)	0.17	−0.037 (−0.096, 0.021)	0.20	−0.029 (−0.082, 0.024)	0.27
HDL-c (mmol/l)	−0.060 (−0.125, 0.000)	0.06	−0.074 (−0.140, −0.01)	0.03	−0.009 (−0.054, 0.073)	0.77	0.034 (−0.026, 0.094)	0.26
LDL-c (mmol/l)	−0.006 (−0.043, 0.031)	0.74	−0.002 (−0.043, 0.039)	0.93	−0.031 (−0.068, 0.006)	0.10	−0.023 (−0.067, 0.022)	0.30

SBP – systolic blood pressure; DBP – diastolic blood pressure.

Model 1 – age and sex adjusted.

Model 2 – as model 1 plus ethnicity, PIR, smoking.

Model 3 – as model 2 plus waist circumference.

Model 4 – as model 3 plus 25(OH)D and adjusted calcium.

*N = 1,193 in all models.

† z-score of log transformed values.

Associations of adjusted calcium with cardiovascular risk factors are presented in Table 3. In age and sex adjusted models calcium was positively associated with all risk factors except with HDL-c and DBP (model 1) and these associations remained when controlling for other potential confounders (models 2 and 3).

**Table 3:** Multivariable associations of calcium with cardiovascular risk factors.

	Model 1 (N = 3,958)		Model 2 (N = 3,958)		Model 3 (N = 3,958)		Model 4 (N = 2,554)	
	Mean change z-score per 1 z-score difference in 25(OH)D (95%CI), p-vale							
SBP (mm/Hg)	0.084 (0.050, 0.119)	<0.001	0.072 (0.036, 0.108)	<0.001	0.064 (0.028, 0.100)	0.001	0.092 (0.040, 0.144)	0.001
DBP (mm/Hg)	0.019 (−0.014, 0.051)	0.25	0.017 (−0.017, 0.050)	0.33	0.007 (−0.029,0.043)	0.69	0.062 (0.007,0.116)	0.03
Fasting glucose (mmol/l)	0.062 (0.022, 0.101)	0.003	0.067 (0.029, 0.106)	0.001	0.055 (0.018, 0.092)	0.005	0.068 (0.016, 0.119)	0.012
2-h glucose† (mmol/l)	0.101 (0.049,0.152)	0.001	0.105 (0.056,0.154)	<0.001	0.078 (0.025,0.130)	0.007	0.083 (0.032,0.133)	0.004
HbA1c (%)	0.098 (0.062, 0.135)	<0.001	0.082 (0.046, 0.118)	<0.001	0.070 (0.036, 0.105)	<0.001	0.090 (0.041, 0.140)	<0.001
Fasting insulin‡ (pmol/l)	0.113 (0.074, 0.151)	<0.001	0.111 (0.071, 0.152)	<0.001	0.074 (0.044, 0.104)	<0.001	0.123 (0.080, 0.167)	<0.001
Fasting triglycerides‡ (mmol/l)	0.096 (0.053, 0.139)	<0.001	0.121 (0.078, 0.165)	<0.001	0.102 (0.063, 0.141)	<0.001	0.136;(0.092,0.179)	<0.001
HDL-c* (mmol/l)	−0.041;(−0.091,0.008)	0.10	−0.043 (−0.094, 0.007)	0.09	−0.019 (−0.070, 0.031)	0.44	−0.022 (−0.067, 0.023)	0.33
LDL-c (mmol/l)	0.056;(0.019,0.093)	0.004	0.059 (0.022, 0.096)	0.003	0.052 (0.014, 0.091)	0.01	0.039 (−0.016, 0.095)	0.16

SBP – systolic blood pressure; DBP – diastolic blood pressure.

Model 1 – age and sex adjusted.

Model 2 – as model 1 plus ethnicity, PIR, smoking.

Model 3 – as model 2 plus waist circumference.

Model 4 – as model 3 plus 25(OH)D.

*N = 2,557 in models 1 to 3 and N = 2,554 in model 4.

† N = 1,193 in all models.

‡ z-score of log transformed values.

Associations observed in the confounder adjusted model (model 3 in tables 1, 2 & 3) were largely unchanged by mutual adjustment for 25(OH)D, PTH and adjusted calcium (model 4 in tables 1, 2, & 3). The only change was to the confounder adjusted association of adjusted calcium with DBP (0.007, 95%CI: −0.029, 0.043) which was strengthened upon adjustment for 25(OH)D and (logged) PTH (0.062 95%CI: 0.007, 0.116). A closer examination showed that PTH was the covariable driving this change. This is not surprising given the lack of evidence of an association between 25(OH)D and adjusted calcium (Pearson's r = −0.01, p-value = 0.49 and Table S1) and between 25(OH)D and DBP (Table 1). Figure S1 presents results from the mutually adjusted models for fasting glucose, insulin and HDL-c.

Of note, results of models 1–3 in Table 1 (25(OH)D as the main exposure) and Table 3 (adjusted calcium as the main exposure, N = 3,958) were not different from those presented when the sample was restricted to participants with PTH measures (N = 2,554). When BMI was included instead of waist circumference, there was no difference to the results presented in model 3 (in Tables 1–3). Moreover, adding a term for physical activity and alcohol consumption did not affect results which is expected as neither was associated with 25(OH)D, PTH or adjusted calcium (Table S1). Finally, excluding participants diagnosed with osteoporosis, taking vitamin D supplementation, participants with elevated fasting glucose or with low eGFR also did not substantially alter results compared to those presented.

## Discussion

We found that 25(OH)D is inversely associated with SBP, fasting insulin, glucose and HbA1c, and positively associated with HDL-c, after adjustment for multiple potential confounders in a general population of adults. These associations were largely unaffected by adjustment for PTH and adjusted calcium. In comparison, there was no strong evidence of associations of PTH with outcomes except for a positive association with DBP and adjusted calcium was positively associated with all cardiovascular risk factors except with DBP and HDL-c, with which no associations were found.

A limited number of studies have previously compared associations of two of 25(OH)D, PTH and calcium with cardiovascular risk factors. In one study of older men (N  =  410) and women (N  =  660) there was no strong evidence of an association of 25(OH)D with the metabolic syndrome and a positive association of PTH with the metabolic syndrome in men only.[19] By contrast, a previous publication using data only from the 2003–2004 NHANES survey (N =  1,654) reported an inverse association of 25(OH)D with metabolic syndrome in both men and women, and also a positive association of PTH with metabolic syndrome in older men (> = 50 years) only.[20] The focus on the metabolic syndrome as opposed to individual cardiovascular risk factors may have resulted in some of the associations that we have found here being missed in these two studies. Similarly to our findings, Zhao et al. found inverse associations of 25(OH)D but not of PTH with measures of insulin resistance in adult participants in NHANES 2003–2006.[11] That study did examine associations with blood pressure or lipids and associations of 25(OH)D were not adjusted for PTH and vice versa.

In the current analysis, associations of 25(OH)D with outcomes persisted when controlling for PTH and adjusted calcium, suggesting that 25(OH)D is associated with cardiovascular risk factors via pathways that are not mediated by the latter. This is in line with results of a prospective study demonstrating an inverse association of 25(OH)D with future hyperglycaemia and insulin resistance, after adjustment for PTH.[9] A recent study examined associations of PTH and adjusted calcium with cardiovascular risk factors in a general population of 1,016 Swedish men and women, 70 years old[5]. In that study, PTH was associated with more cardiovascular risk factors than adjusted calcium, in contrast to our findings. However, a meta-analysis of randomised controlled trials of calcium supplementation found that calcium supplementation was associated with an increased risk of myocardial infarction, stroke, or sudden death.[21]

We found strong evidence that PTH is positively associated with DBP and weaker evidence of a positive association with SBP. One previous cross sectional study found positive associations of PTH with diastolic and systolic blood pressure and with hypertension, independent of potential confounding factors and 25(OH)D.[10] In that study 25(OH)D was not associated with blood pressure variation or hypertension. Consistent with our findings, a prospective study using data on sub-samples from the Health Professional's and Nurses' Health studies found independent inverse associations of 25(OH)D with incident hypertension,[22] and positive associations of PTH with incident hypertension (the latter using data only from the male Health Professional study).[23]

Given that chronic kidney disease adversely affects vitamin D status and is associated with cardiovascular risk,[24] we repeated analyses excluding participants with moderate or severe chronic kidney disease. Results were unchanged to those presented suggesting that observed associations are not driven by participants with chronic kidney disease.

The main limitation of this analysis is that we cannot discern temporality due to the cross sectional nature of the data and therefore we cannot infer causality between levels of 25(OH)D, PTH and adjusted calcium and cardiovascular disease risk factors, though findings from prospective studies support a direction of effect from 25(OH)D to hypertension and impaired glucose metabolism.[8], [9], [22] We were unable to adjust for seasonality of blood taking but seasonality is unlikely to confound associations, since we would not expect it to be associated with the outcomes we examined here, other than through its effect on circulating 25(OH)D. However, without adjustment for seasonality the associations we have examined may be an underestimate of the true associations.[25] Finally, drifts in the 25(OH)D assay performance over time, likely due to reagent and calibration lot changes in the reformulated DiaSorin assay, have been noted[26]. However, as recommended by the National Center for Health Statistics, we combined data from three cycles in order to obtain stable estimates.

Taken together our findings suggest that the associations of vitamin D, calcium and PTH with incident cardiovascular disease[1]–[4], [27] represent different underlying associations with cardiovascular risk factors, with vitamin D and calcium likely acting through pathways involving glucose and lipid metabolism and PTH having a more important effect via blood pressure. It has been suggested that vitamin D status affects both beta cell function and insulin release and sensitivity, both being calcium dependent pathways, as well as inflammatory processes.[9], [28] It has also been suggested that PTH affects blood pressure through a proliferative effect on vascular smooth muscle cells.[5], [29]

To summarize, in a large general population sample of adults without diabetes or coronary heart disease, we found lower levels of 25(OH)D to be associated with higher levels of fasting glucose, insulin and SBP and lower levels of HDL-c. PTH was positively associated with DBP, but not other cardiovascular risk factors and adjusted calcium was associated with all outcomes except DBP and HDL-c. These findings suggest that the recently reported prospective associations of 25(OH)D and PTH with cardiovascular disease occur through different underlying associations, with proximal risk factors. Additional research should examine whether 25(OH)D, calcium and PTH are independently associated with cardiovascular disease and its risk factors in prospective studies. Finally, genetic variants that are robustly (and independently) associated with variation in 25(OH)D, PTH and calcium levels could be used as instrumental variables[30] to examine their causal effects on cardiovascular risk factors and events.

## Supporting Information

Figure S1 Change in z-score of fasting glucose, insulin and HDL-c per 1 z-score difference in 25(OH)D, PTH and calcium, adjusted for confounders and mutual adjustment (equivalent to model 4 in Tables 1–3).
(0.08 MB DOC)

Table S1 Characteristics (% or mean and 95%CI) across fifths of the 25(OH)D distribution. N  =  3,958.
(0.12 MB DOC)
