# Supplementary material for: Associations of Serum 25-Hydroxyvitamin D, Parathyroid Hormone and Calcium with Cardiovascular Risk Factors: Analysis of 3 NHANES Cycles (2001–2006)
Source: PLoS One. 2010 Nov 9;5(11):e13882. doi: 10.1371/journal.pone.0013882 (PMC2976699; doi:10.1371/journal.pone.0013882)
Supplement: Figure S1 — Change in z-score of fasting glucose, insulin and HDL-c per 1 z-score difference in 25(OH)D, PTH and calcium, adjusted for confounders and mutual adjustment (equivalent to model 4 in Tables 1– 3). (0.08 MB DOC) [file pone.0013882.s001.doc]

Figure S1. Change in z-score of fasting glucose, insulin and HDL-c per 1 z-score difference in 25(OH)D, PTH and calcium, adjusted for confounders and mutual adjustment (equivalent to model 4 in Tables 1-3)

| 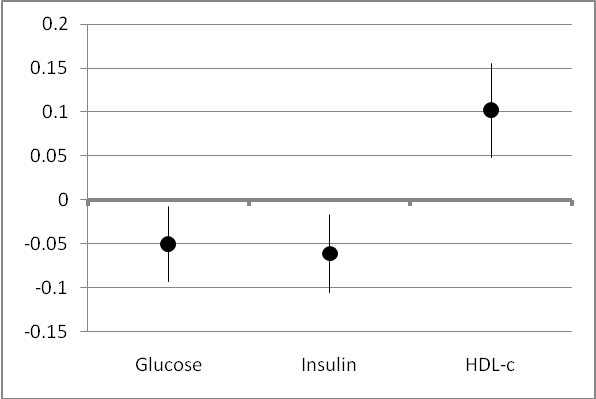  Panel a. 25(OH)D. | 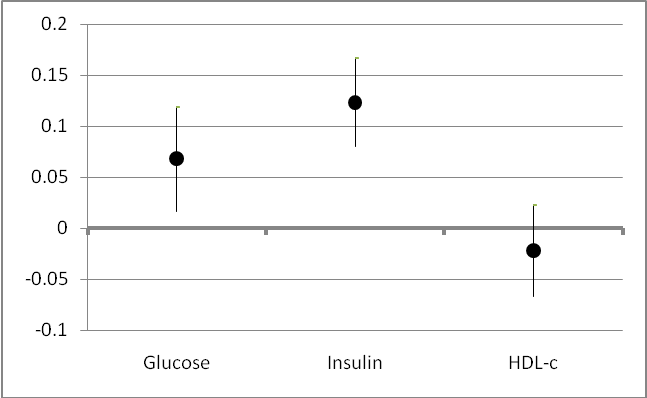  Panel c. Calcium. |
| --- | --- |
| 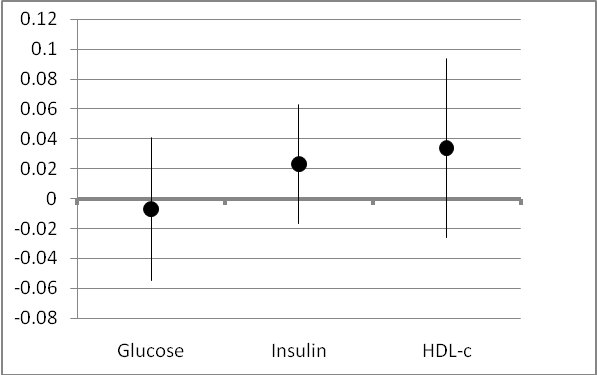  Panel b. PTH | Glucose SD- 0.37 mmol/l  Insulin SD – 0.8 pmol/l  HDL-C SD – 0.4 mmol/l |
