# Supplementary material for: Associations of Serum 25-Hydroxyvitamin D, Parathyroid Hormone and Calcium with Cardiovascular Risk Factors: Analysis of 3 NHANES Cycles (2001–2006)
Source: PLoS One. 2010 Nov 9;5(11):e13882. doi: 10.1371/journal.pone.0013882 (PMC2976699; doi:10.1371/journal.pone.0013882)
Supplement: Table S1 — Characteristics (% or mean and 95%CI) across fifths of the 25(OH)D distribution. N = 3,958. (0.12 MB DOC) [file pone.0013882.s002.doc]

Supplementary Table 1. Characteristics (% or mean and 95%CI) across fifths of the 25(OH)D distribution. N = 3,958

|  | **Lowest fifth**  **<35.0** **nmol/l** N=862 | **2^nd^ fifth**  **35.0-48.9 nmol/l**  N=746 | **3^rd^ fifth**  **49.0-59.9 nmol/l**  N=883 | **4^th^ fifth**  **60.0-73.9 nmol/l**  N=699 | **Top fifth**  **≥74.0 nmol/l**  N=768 | **p-value linear trend** |
| --- | --- | --- | --- | --- | --- | --- |
| Age | 40.8 (38.6, 43.0) | 41.3 (39.2, 43.3) | 42.2 (40.0, 44.3) | 44.3 (42.5, 46.1) | 43.0 (41.3, 44.6) | <0.001 |
| Male | 41.2 (37.1, 45.5) | 48.3 (43.6, 53.0) | 51.4 (48.0, 54.8) | 52.8 (48.4, 57.1) | 48.9 (44.5, 53.3) | 0.01 |
| Ethnicity  White  Black  Mexican  Other | 42.5 (36.3, 49.0)  34.7 (28.6, 41.4)  8.3 (5.5, 12.2)  12.2 (7.9, 18.2) | 68.8 (62.3, 74.6)  11.0(8.5, 14.1)  8.3 (5.9, 11.4)  11.2 (8.1, 6.8) | 80.6 (77.0, 83.7)  5.8 (4.3, 7.9)  6.4 (5.0, 8.3)  6.9 (5.0, 9.5) | 88.2 (84.4, 91.1)  2.7 (1.7, 4.3)  4.5 (3.1, 6.6)  4.6 (3.1, 6.8) | 91.9 (88.5, 94.3)  1.3 (0.1, 1.9)  2.5 (1.7, 3.8)  4.6 (2.8, 7.4) | <0.001  <0.001  <0.001  <0.001 |
| Household above poverty threshold | 85.2 (81.7, 88.2) | 90.5 (87.4, 92.9) | 90.6 (87.9, 92.8) | 94.3 (92.2, 95.8) | 94.0 (92.3, 95.3) | <0.001 |
| Ever smoked | 45.8 (41.3, 50.5) | 48.3 (43.4, 53.3) | 52.2 (47.7, 56.7) | 54.9 (49.9, 59.8) | 53.7 (48.8, 58.6) | 0.05 |
| Top third of alcohol intake  N=2,710 | 13.6 (10.4, 17.5) | 15.7 (11.8, 20.5) | 15.7 (11.8, 20.5) | 15.6 (12.2, 19.8) | 14.2 (11.7, 17.2) | 0.41 |
| Physical activity (MET, hours per day)  N=2,736 | 3.1 (2.0, 4.2) | 3.9 (2.6, 5.2) | 3.4 (2.2, 4.7) | 3.1 (1.9, 4.2) | 3.8 (2.5, 5.0) | 0.63 |
| Osteoporosis | 2.3 (1.3, 4.0) | 1.9 (0.9, 4.0) | 1.7 (1.1, 2.6) | 2.6 (1.7, 3.9) | 3.2 (2.1, 4.7) | 0.26 |
| Vitamin D supplementation | 19.9 (16.7, 23.6) | 33.9 (29.9, 38.2) | 43.2 (38.2, 48.5) | 47.0 (42.2, 51.8) | 53.4 (49.6, 57.2) | <0.001 |
| BMI (kg/m^2^) | 29.0 (28.2, 29.9) | 27.9 (26.8, 29.1) | 26.3 (25.5, 27.1) | 26.0 (25.1, 26.9) | 25.4 (24.7, 26.1) | <0.001 |
| Waist circumference (cm) | 88.5 (86.3, 90.7) | 86.6 (83.8, 89.3) | 82.9 (80.8, 84.9) | 82.5 (80.4, 84.7) | 81.0 (79.1, 82.8) | <0.001 |
| SBP (mm/Hg) | 101.5 (99.4, 103.5) | 97.7 (96.0, 99.4) | 97.0 (95.1, 98.8) | 95.5 (94.0, 97.1) | 95.7 (93.9, 97.5) | <0.001 |
| DBP (mm/Hg) | 69.2 (67.3, 71.2) | 68.6 (66.9, 70.4) | 68.5 (66.6, 70.4) | 68.1 (66.2, 70.1) | 68.4 (66.1, 70.7) | 0.28 |
| Fasting glucose (mmol/l) | 4.70 (4.60, 4.80) | 4.64 (4.56, 4.73) | 4.60 (4.53, 4.67) | 4.57 (4.48, 4.67) | 4.51 (4.43, 4.59) | 0.001 |
| 2-h glucose† (mmol/l)  N=1,125 | 4.15 (3.81, 4.51) | 3.95 (3.69, 4.24) | 3.81 (3.56, 4.07) | 3.79 (3.48, 4.13) | 3.65 (3.45, 3.87) | <0.001 |
| HbA1c (%) | 4.96 (4.91, 5.01) | 4.86 (4.81, 4.91) | 4.84 (4.80, 4.88) | 4.83 (4.77, 4.88) | 4.77 (4.73, 4.82) | <0.001 |
| Fasting insulin‡ (pmol/l) | 49.0 (44.3, 54.2) | 45.9 (40.4, 52.1) | 36.2 (32.6, 40.1) | 33.5 (30.0, 37.6) | 32.3 (28.6, 36.5) | <0.001 |
| Fasting triglycerides‡ (mmol/l) | 0.87 (0.80, 0.94) | 0.91 (0.83, 1.01) | 0.89 (0.81, 0.97) | 0.86 (0.80, 0.93) | 0.84 (0.78, 0.91) | 0.06 |
| HDL-c* (mmol/l)  N=2,564 | 1.39 (1.34, 1.45) | 1.40 (1.34, 1.46) | 1.48 (1.43, 1.54) | 1.49 (1.41, 1.56) | 1.54 (1.47, 1.61) | <0.001 |
| LDL-c (mmol/l) | 2.53 (2.38, 2.69) | 2.52 (2.38, 2.65) | 2.55 (2.44, 2.67) | 2.54 (2.36, 2.72) | 2.56 (2.41, 2.71) | 0.56 |
| PTH*‡ (ng/l)  N=2,554 | 34.79 (32.48, 37.26) | 30.10 (28.20, 32.13) | 28.43 (26.23, 30.82) | 27.64 (25.41, 30.07) | 24.49 (22.50, 26.66) | <0.001 |
| Adjusted calcium (mmol/l) | 2.30 (2.29, 2.31) | 2.29 (2.28, 2.30) | 2.29 (2.28, 2.30) | 2.28 (2.27, 2.29) | 2.29 (2.28, 2.31) | 0.31 |

DBP – diastolic blood pressure; SBP – systolic blood pressure

* 2003-4 and 2005-6 surveys† 2005-6 survey

‡ geometric mean

Estimates are centred for age and sex to ensure any associations are not explained by age and sex.

Supplementary Table 2. Characteristics (% or mean and 95%CI) across fifths of the PTH distribution. N = 2,554

|  | **Lowest fifth**  **<=6.39 (geometric mean ng/l)** N=514 | **2^nd^ fifth**  **28.00-36.00**  N= **535** | **3^rd^ fifth**  **36.10-44.00**  N= **505** | **4^th^ fifth**  **44.10-56.99**  N= **521** | **Top fifth**  **>=57.00**  N= **479** | **p-value linear trend** |
| --- | --- | --- | --- | --- | --- | --- |
| Age | 39.9 (38.4, 41.5) | 43.0 (41.2, 44.8) | 45.2 (43.1, 47.3) | 48.5 (46.4, 50.6) | 51.8 (49.1, 54.5) | < 0.001 |
| Male | 54.7 (49.3, 60.0) | 50.8 (45.7, 55.7) | 45.3 (41.3, 49.3) | 51.4 (44.6, 58.1) | 42.7 (35.7, 50.0) | 0.02 |
| Ethnicity  White  Black  Mexican  Other | 81.5 (76.8, 85.5)  6.5 (4.7, 9.2)  4.0 (2.4, 6.4)  7.8 (5.3, 11.5) | 76.7 (71.0, 81.5)  7.9 (5.5, 11.1)  6.2 (3.9, 9.8)  8.6 (5.9, 12.5) | 76.6 (70.4, 81.8)  8.8 (5.9, 12.8)  5.2 (3.2, 8.1)  8.9 (5.9, 13.2) | 76.0 (68.8, 82.0)  10.2 (7.1, 14.3)  6.1 (3.9, 9.6)  6.8 (4.0, 11.3) | 70.9 (64.0, 76.9)  13.2 (9.6, 17.9)  7.3 (4.1, 12.5)  6.8 (4.6, 10.1) | < 0.001  < 0.001  < 0.001  0.89 |
| Household above poverty threshold | 92.0 (89.0, 94.3) | 89.3 (86.5, 91.7) | 92.1 (88.6, 94.5) | 93.0 (90.5, 94.9) | 90.7 (87.2, 93.2) | 0.03 |
| Ever smoked | 58.0 (52.5, 63.2) | 51.4 (46.5, 56.1) | 49.8 (44.8, 54.8) | 50.4 (44.9, 55.9) | 44.3 (38.1, 50.8) | <0.001 |
| Top third of alcohol intake  N=1,750 | 21.6 (17.0, 27.0) | 19.0 (14.2, 24.9) | 14.7 (11.6, 18.5) | 12.7 (9.1, 17.4) | 7.1 (4.8, 10.3) | 0.18 |
| Physical activity (MET, hours per day)  N=1,705 | 2.6 (1.3, 3.9) | 3.3 (2.0, 4.5) | 2.6 (1.4, 3.9) | 1.9 (0.3, 3.4) | 3.5 (1.7, 5.3) | 0.97 |
| Osteoporosis | 0.9 (0.4, 2.2) | 2.1 (1.2, 3.8) | 2.1 (1.0, 4.4) | 2.0 (1.2, 3.3) | 4.9 (2.5, 9.1) | 0.88 |
| Vitamin D supplementation | 43.3 (36.8, 50.0) | 37.0 (32.4, 41.7) | 41.9 (35.7, 48.5) | 46.1 (39.9, 52.4) | 38.2 (32.5, 44.3) | 0.007 |
| BMI (kg/m^2^) | 26.1 (25.2, 27.0) | 27.5 (26.4, 28.6) | 28.9 (27.5, 30.4) | 29.6 (28.3, 31.0) | 30.2 (28.6, 31.8) | < 0.001 |
| Waist circumference (cm) | 82.7 (80.5, 85.0) | 86.0 (83.4, 88.7) | 89.8 (86.4, 93.1) | 91.6 (88.3, 94.8) | 91.7 (88.0, 95.4) | < 0.001 |
| SBP (mm/Hg) | 98.1 (96.2, 100.0) | 99.1 (97.2, 100.8) | 99.8 (97.9, 101.7) | 101.3 (98.5, 104.1) | 103.1 (100.2, 106.1) | 0.004 |
| DBP (mm/Hg) | 67.2 (65.1, 69.3) | 68.7 (66.4, 71.0) | 69.8 (67.5, 72.2) | 71.4 (68.7, 74.3) | 70.9 (67.9, 74.0) | < 0.001 |
| Fasting glucose (mmol/l) | 4.56 (4.47, 4.66) | 4.61 (4.52, 4.71) | 4.66 (4.58, 4.74) | 4.66 (4.58, 4.74) | 4.70 (4.59, 4.82) | 0.01 |
| 2-h glucose† (mmol/l)  N=1,125 | 3.66 (3.44, 3.88) | 3.99 (3.73, 4.27) | 4.12 (3.90, 4.36) | 4.15 (3.74, 4.61) | 4.27 (3.95, 4.61) | < 0.001 |
| HbA1c (%) | 4.81 (4.76, 4.87) | 4.85 (4.79, 4.90) | 4.87 (4.81, 4.93) | 4.87 (4.82, 4.93) | 4.92 (4.85, 4.99) | 0.004 |
| Fasting insulin‡ (pmol/l) | 35.9 (30.8, 41.6) | 43.9 (37.8, 51.1) | 48.0 (41.4, 55.7) | 52.2 (44.9, 60.7) | 57.2 (49.1, 66.6) | < 0.001 |
| Fasting triglycerides‡ (mmol/l) | 0.87 (0.79, 0.95) | 0.87 (0.79, 0.95) | 0.88 (0.79, 0.99) | 0.93 (0.82, 1.04) | 0.88 (0.78, 1.00) | 0.32 |
| HDL-c* (mmol/l) | 1.47 (1.42, 1.53) | 1.46 (1.41, 1.52) | 1.44 (1.36, 1.51) | 1.42 (1.34, 1.49) | 1.42 (1.34, 1.49) | 0.08 |
| LDL-c (mmol/l) | 2.54 (2.37, 2.71) | 2.49 (2.28, 2.69) | 2.61 (2.44, 2.79) | 2.62 (2.43, 2.81) | 2.48 (2.30, 2.67) | 0.51 |
| 25 (OH)D (nmol/l) | 60.87 (53.85, 67.89) | 55.01 (46.20, 63.82) | 47.98 (39.41, 56.54) | 46.23 (36.43, 56.03) | 35.75 (25.36, 46.13) | < 0.001 |
| Adjusted calcium (mmol/l) | 2.32 (2.30, 2.33) | 2.31 (2.29, 2.32) | 2.30 (2.28, 2.31) | 2.29 (2.28, 2.31) | 2.29, (2.27, 2.30) | < 0.001 |

All data from surveys 2003-4 and 2005-6 only (when PTH was assayed)

DBP – diastolic blood pressure; SBP – systolic blood pressure

* 2003-4 and 2005-6 surveys

† 2005-6 survey only

‡ geometric mean

Estimates are centred for age and sex to ensure any associations are not explained by age and sex.

Supplementary Table 3. Characteristics (% or mean and 95%CI) across fifths of the adjusted calcium distribution. N = 3,958

|  | **Lowest fifth**  **<2.25** **mmol/l** N=801 | **2^nd^ fifth**  **2.26-2.29 mmol/l**  N=841 | **3^rd^ fifth**  **2.30-2.33 mmol/l**  N=733 | **4^th^ fifth**  **2.34-2.38 mmol/l**  N=836 | **Top fifth**  **≥2.39 mmol/l**  N=747 | **p-value linear trend** |
| --- | --- | --- | --- | --- | --- | --- |
| Age | 44.6 (41.7, 47.5) | 43.6 (40.9, 46.3) | 45.5 (42.0, 48.9) | 46.0 (42.7, 49.4) | 46.2 (43.6, 48.7) | 0.07 |
| Male | 53.3 (49.5, 57.0) | 53.8 (50.7, 56.9) | 50.3 (45.5, 55.2) | 48.5 (44.8, 52.2) | 36.7 (31.5, 42.2) | <0.001 |
| Ethnicity  White  Black  Mexican  Other | 77.5 (72.6, 81.8)  3.5 (2.3, 5.5)  8.0 (5.9, 10.8)  10.4 (7.8, 13.7) | 78.1 (73.8, 82.0)  8.0 (6.0, 10.5)  6.0 (4.5, 7.9)  7.5 (5.4, 10.3) | 80.6 (76.0, 84.5)  7.4 (5.3, 10.2)  6.1 (4.5, 8.2)  5.5 (3.8, 8.0) | 75.8 (70.3, 80.6)  11.0 (8.0, 14.9)  5.0 (3.3, 7.4)  7.7 (4.9, 12.0) | 74.8 (67.7, 80.8)  16.8 (12.4, 22.4)  2.9 (1.5, 5.7)  5.1 (3.4, 7.7) | 0.03  <0.001  0.003  0.03 |
| Household above poverty threshold | 91.8 (89.1, 93.9) | 91.2 (89.0, 93.0) | 91.2 (88.7, 93.2) | 92.3 (90.1, 94.1) | 90.3 (86.8, 92.9) | 0.14 |
| Ever smoked | 47.8 (42.2, 53.6) | 52.4 (47.6, 57.2) | 50.0 (45.4, 54.6) | 54.2 (49.3, 58.9) | 51.6 (46.8, 56.3) | 0.26 |
| Top third of alcohol intake  N=2,710 | 15.5 (13.1, 18.4) | 16.6 (13.4, 20.4) | 15.0 (12.3, 18.3) | 16.0 (13.0, 20.0) | 11.8 (8.8, 15.7) | 0.18 |
| Physical activity (MET, hours per day)  N=2,736 | 3.5 (2.2, 4.8) | 3.2 (1.8, 4.6) | 3.7 (2.7, 4.7) | 3.6 (2.5, 4.8) | 3.5 (2.3, 4.7) | 0.70 |
| Osteoporosis | 1.5 (0.9, 2.4) | 1.9 (1.1, 3.3) | 1.6 (0.9, 2.8) | 3.4 (2.0, 5.7) | 3.8 (2.6, 5.5) | 0.27 |
| Vitamin D supplementation | 43.0 (39.1, 47.0) | 40.2 (35.8, 44.8) | 40.5 (35.9, 45.3) | 37.5 (33.5, 41.7) | 44.7 (39.9, 49.6) | 0.01 |
| BMI (kg/m^2^) | 26.6 (25.8, 27.5) | 26.9 (26.1, 27.7) | 27.3 (26.4, 28.2) | 27.3 (26.4, 28.2) | 27.4 (26.4, 28.4) | 0.02 |
| Waist circumference (cm) | 83.0 (80.8, 85.1) | 84.1 (82.1, 86.0) | 85.4 (83.4, 87.4) | 85.5 (83.3, 87.8) | 85.5 (83.0, 88.0) | 0.003 |
| SBP (mm/Hg) | 96.5 (94.6, 98.4) | 97.2 (95.6, 98.9) | 97.5 (95.6, 99.5) | 98.9 (97.4, 100.4) | 101.3, 99.1, 103.4) | <0.001 |
| DBP (mm/Hg) | 68.7 (66.8, 70.6) | 68.4 (66.3, 70.4) | 68.9 (67.2, 70.5) | 68.7 (66.8, 70.5) | 69.0 (67.0, 70.9) | 0.61 |
| Fasting glucose (mmol/l) | 4.58 (4.52, 4.65) | 4.58 (4.51, 4.66) | 4.64 (4.56, 4.71) | 4.60 (4.52, 4.68) | 4.73 (4.63, 4.83) | 0.005 |
| 2-h glucose† (mmol/l)  N=1,125 | 3.78 (3.49, 4.13) | 3.78 (3.55, 4.01) | 3.82 (3.56, 4.11) | 3.86 (3.59, 4.16) | 4.23 (3.99, 4.49) | 0.001 |
| HbA1c (%) | 4.82 (4.78, 4.85) | 4.82 (4.77, 4.87) | 4.88 (4.84, 4.93) | 4.88 (4.84, 4.92) | 74.95 (4.90, 5.01) | <0.001 |
| Fasting insulin‡ (pmol/l) | 35.8 (32.3, 39.6) | 37.7 (33.9, 41.8) | 41.0 (36.8, 45.7) | 42.1 (37.4, 47.3) | 46.0 (39.5, 53.6) | <0.001 |
| Fasting triglycerides‡ (mmol/l) | 0.83 (0.76, 0.91) | 0.84 (0.78, 0.90) | 0.91 (0.84, 0.99) | 0.92 (0.84, 1.01) | 0.93, 0.85, 1.01) | <0.001 |
| HDL-c* (mmol/l)  N=2,557 | 1.49 (1.43, 1.55) | 1.46 (1.41, 1.52) | 1.43 (1.37, 1.49) | 1.46 (1.39, 1.52) | 1.45 (1.37, 1.53) | 0.24 |
| LDL-c (mmol/l) | 2.45 (2.30, 2.60) | 2.53 (2.38, 2.67) | 2.60 (2.45, 2.76) | 2.59 (2.47, 2.70) | 2.59 (2.46, 2.72) | 0.013 |
| 25(OH)D (nmol/l) | 56.0 (52.2, 59.9) | 56.3 (52.3, 60.3) | 58.6 (54.1, 63.1) | 56.0 (51.9, 60.1) | 55.7 (50.0, 61.4) | 0.89 |
| PTH*‡ (ng/l)  N=2,554 | 32.2 (30.0, 34.6) | 29.2 (27.4, 31.1) | 28.8 (26.5, 31.3) | 28.5 (26.4, 30.8) | 26.1 (24.0, 28.3) | <0.001 |

DBP – diastolic blood pressure; SBP – systolic blood pressure

* 2003-4 and 2005-6 surveys

† 2005-6 survey

‡ geometric mean

Estimates are centred for age and sex to ensure any associations are not explained by age and sex.
